# Colorectal Cancer Prevention in Eastern European (EaP) Countries: A Policy Analysis

**DOI:** 10.1093/eurpub/ckac131.002

**Published:** 2022-10-25

**Authors:** M Nonikashvili, M Kereselidze, T Beruchashvili

**Affiliations:** Public Health and Healthcare Management, School of Health Sciences, University of Georgia, Tbilisi, Georgia

## Abstract

**Background:**

Colorectal cancer (CRC) is a critical public health issue in Central and Eastern European Countries, where it is the second leading cause of female cancer deaths. In this study, we aimed to identify the presence of CRC policies in EaP Countries and analyze the content of CRC policies for comprehensiveness.

**Methods:**

We conducted a scoping review based on the methodological framework suggested by Arksey and O'Malley. We searched for publicly-available policy documents from Armenia, Azerbaijan, Belarus, Georgia, Moldova and Ukraine. We outline each country’s prevention approaches and activities based on World Health Organization (WHO) guidelines for CRC screening.

**Results:**

Most countries had at least one policy addressing some aspect of colorectal cancer prevention. Primary and secondary prevention were most commonly addressed, and certain details such as healthy lifestyle campaigns, target age and screening method (gFOBT, FIT or colonoscopy) were frequently mentioned in these policies. Policies to implement or pilot population-based CRC screening program have been adopted only by Georgia. Our analysis revealed the urgent need to improve the availability and use of colorectal cancer screening among those countries.

**Conclusions:**

These country experiences suggest that while prevention approaches can promote the best strategies to carry out screening programs, there is no standardized approach to screening for CRC.

**Key messages:**

• Further research is urgently needed to better understand CRC screening needs of adults in EaP.

• Necessity of CRC screening irrespective of need will help make shift from curative to preventive cancer care.

